# Outcomes of cataract surgery in a rural and urban south Indian population

**DOI:** 10.4103/0301-4738.62648

**Published:** 2010

**Authors:** Lingam Vijaya, Ronnie George, Rashima A, Prema Raju, Hemamalini Arvind, Mani Baskaran, Ramesh S Ve

**Affiliations:** Glaucoma Project, Vision Research Foundation, Sankara Nethralaya, Chennai, India

**Keywords:** Cataract surgery, epidemiology, India, population, visual impairment

## Abstract

**Purpose:**

To assess the visual outcome after cataract surgery in a south Indian population.

**Materials and Methods:**

Population-based cross-sectional study of subjects aged 40 years or more. Three thousand nine hundred and twenty-four rural subjects from 27 contiguous villages and 3850 urban subjects from five randomly selected divisions were studied. All subjects underwent a comprehensive ophthalmic examination that included visual acuity, refraction, slit-lamp biomicroscopy, applanation tonometry, gonioscopy, and dilated retinal examination.

**Statistical Analysis:**

Chi square test, *t* test and multivariate analysis were used.

**Results:**

Five hundred and twenty-eight (216 males, 312 females, 781 eyes) rural subjects (13.5%, 95% confidence interval (CI) 12.4% to 14.6%) and 406 (197 males, 209 females, 604 eyes) urban subjects (10.5%, 95% CI 9.6-11.5%) had undergone cataract surgery. Outcome of cataract surgery was defined based on visual acuity. Using best-corrected visual acuity for classification, the single most important cause for visual impairment was cystoid macular edema in the aphakic group and posterior capsule opacification in the pseudophakic group. Aphakia (visual acuity of <20/60 to ≤20/400 - odds ratio (OR) 1.8; 95% CI 1.3 to 2.6%, visual acuity of <20/400 - OR 6.2; 95% 4.0 to 9.8%), rural residence (visual acuity of <20/60 to ≤20/400 - OR 3.2; 95% CI 2.2 to 4.5% and visual acuity of <20/400 - OR OR 3.5; 95% CI 2.3 to 5.5%) were associated with visual impairment. The urban cataract-operated population had significantly more pseudophakics (*P* < 0.001), men (*P* = 0.02) and literates (*P* < 0.001). In the rural group the prevalence of cataract surgery (13.5% vs. 10.5%, *P* < 0.001) and number of people that had undergone cataract surgery within three years prior to examination (*P* < 0.001) were significantly greater. In 30% of rural and 16% of urban subjects uncorrected refraction was the cause of visual impairment.

**Conclusions:**

Surgery-related complications were major causes for visual acuity of <20/60.

Cataract remains the leading cause of blindness in India.[1–4] The main emphasis of the National Program for Control of Blindness (NPCB) in India was on cataract blindness control.[5] As a result, the number of cataract surgeries performed increased from 1.2 million/year in 1992 to 3.86 million/year in the year 2003.[6] In the “Vision 2020: The Right to Sight” initiative the target was to perform 21.1 million cataract surgeries during 2002-07 with 80% intraocular lens implantation.[7] Even though the cataract surgical targets are met, poor outcomes of cataract surgery is a major problem in developing countries.[8–11] The Chennai glaucoma study (CGS) is a population-based cross-sectional study in a rural and an urban south Indian population aged ≥40 years.[12] The purpose of this study was to report the visual outcome of cataract surgery and associated factors that influenced visual outcomes in the population we studied. We further compared outcomes between the rural and urban populations.

## Materials and Methods

The detailed methodology of the CGS has been published earlier.[12] This study was approved by the Institutional Ethics Review Board and was performed in accordance with the tenets of the Declaration of Helsinki. Written informed consent was obtained from eligible subjects. All subjects underwent a complete ophthalmic examination at the base hospital. Examination consisted of measuring the best-corrected visual acuity using the modified ETDRS chart, applanation tonometry, gonioscopy, grading of lens opacities using LOCS II,[13] stereoscopic evaluation of the optic nerve head and macula at the slit-lamp using a +78 diopter lens, a detailed retinal examination with a binocular indirect ophthalmoscope, and optic disc and fundus photography.

We measured the presenting visual acuity using logarithm of minimum angle of resolution (logMAR) 4-meter charts at 4 meters, those unable to read the top line of the chart were tested at 1 meter. Landolt's C chart was used for those who could not read English. Monocular visual acuity was recorded with the current spectacle prescription, if any. Pinhole acuity was assessed in eyes with presenting visual acuity less than 20/20 (logMAR 0.0) to estimate the end point of subjective refraction. Streak retinoscopy and subjective refraction were performed on all subjects. The best-corrected visual acuity was ascertained and the value recorded. If the visual acuity could not be measured we used the following tests sequentially: Counting fingers, hand movements and light perception. In cataract-operated eyes the probable method of surgery performed, the presence or absence of intraocular lens (IOL), type of IOL (anterior and posterior chamber) and possible cataract-related complications were documented. The visual outcome of eyes that had undergone cataract surgery was assessed based on the presenting and best-corrected visual acuity. We also reported visual impairment of subjects based on visual acuity in the better eye (irrespective of lens status). We classified people with at least primary education as literate and people with no formal education as illiterate.[14] Data analysis was carried out using SPSS (SPSS Inc., Chicago, IL). Significance was assessed at the *P* < 0.05 level for all parameters. Multivariate analysis for age, gender, residence (rural or urban), duration from surgery and literacy was done after adjusting for age (the age group of 40-49 years was used as the reference age group). In bilaterally operated persons only the right eye was included for multivariate analysis.

## Results

A total of 9600 (4800 each) subjects were enumerated in the urban and rural arms of the study. Of this, 3924 rural subjects and 3850 urban subjects participated in the study. In the demographic profile there were no differences between the urban and the rural groups in terms of gender distribution, participation and non-participation rates. However, the mean age of the urban population was significantly older than that of the rural population. This was similar among both participants (54.8 ± 10.6 years vs. 53.8 ± 10.6 years; *P* < 0.001) and non-participants (53.8 ± 10.9 years vs. 52.5 ± 10.5 years; *P* < 0.001).

Demographic information is provided in Table 1. In the rural population 528 (216 males, 312 females) out of 3924 subjects had undergone cataract surgery. The prevalence of aphakia/pseudophakia was 13.5% (95% CI 12.4-14.6%). Seven hundred and eighty-one eyes of 528 subjects were analyzed in this study. In 13 of the 781 cataract-operated eyes the anterior segment details could not be visualized, due to gross distortion of the eyeball anatomy, following cataract surgery. In the urban population, 406 (197 males, 209 females) of 3924 subjects had undergone cataract surgery. The prevalence of aphakia/pseudophakia was 10.5% (95% CI 9.6-11.5%). Six hundred and four eyes of 406 subjects were analyzed in this study. The mean age of those who had undergone cataract surgery was significantly older than the phakic study population in both groups (*P* < 0.001).

**Table 1:** Demographics of cataract surgery group- rural and urban

Age group in yrs	Number Rural/Urban(%)	Cataract surgery prevalence (%) Rural/Urban	Gender Male:Female Rural/Urban	Literate Male: Female Rural/Urban	Illiterate Male: Female Rural/Urban	Pseudophakia* Male:Female Rural†/Urban	Aphakia* Male:Female Rural†/Urban
40-49	16 (3.0)	1.0	3:13	2:0	1:13	2:11	1:2
	25 (6.2)	1.8	9:16	9:12	0:4	6:14	3:2
50-59	85 (16.1)	8.6	29:56	18:17	11:39	16:40	13:16
	54 (13.3)	4.8	20:34	17:24	3:10	17:26	3:8
60-69	230 (43.6)	25.8	80:150	39:39	41:111	55:89	23:61
	168 (41.4)	18.5	87:81	80:65	7:16	72:64	15:17
70-79	168 (31.8)	41.6	82:86	41:19	41:67	43:37	39:49
	134 (33.0)	37.6	67:67	57:35	10:32	44:42	23:25
≥80	29 (5.5)	50.9	22:7	16:2	6:5	6:3	16:4
	25 (6.2)	51.0	14:11	11:7	3:4	7:8	7:3
Total	528		216:312	116:77	100:235	122:180	92:132
	406		197:209	174:143	23:66	146:154	55:106

* -In subjects with one eye pseudophakia and other eye aphakia division was done based up on right eye status;

† -In rural population 2 subjects were phakic in theright eye and had cataract surgery in the left eye with complications that prevented categorization as either pseudophakia or aphakia

Table 2 provides details of the rural and urban cataract- operated population. There was no difference in the proportion of persons who had undergone unilateral or bilateral cataract surgery between both groups. However, the urban cataract-operated population had significantly more pseudophakics (*P* < 0.001), men (*P* = 0.02) and literates (*P* < 0.001). In the rural group the prevalence of cataract surgery (13.5% vs. 10.5%, *P* < 0.001) and number of persons who had undergone cataract surgery within three years prior to examination (*P* < 0.001) was significantly greater. The power of the study to detect the difference in the visual outcome between aphakia and pseudophakia in both populations was 99.8%.

**Table 2:** Demographic differences in the rural and urban cataract-operated population*

	Rural population	Urban population	*P* value
Operated in one eye number (%)	275 (52.1)	208 (51.2)	0.85
Operated in both eyes number (%)	253 (47.9)	198 (48.8)	0.85
Pseudophakia number of eyes (%)	426 (55.4)	440 (72.8)	<0.001
Aphakia number of eyes (%)	342 (44.5)	164 (27.2)	<0.001
Males	216 (40.9)	197 (48.5)	0.02
Females	312 (59.1)	209 (51.5)	
Mean age in years ± SD	64.9 ± 8.7	66.1 ± 9.4	0.044
Duration from surgery†			
<3 years	333 (63.1)	207 (51.0)	<0.001
>3 years	191 (36.1)	199 (49.0)	<0.001
Literates	193 (36.6)	317 (78.1)	<0.001
Illiterates	335 (63.4)	89 (21.9)	
Prevalence of cataract	528 (13.4)	406 (10.5)	<0.001
surgery - Number (%)			

* -Chi square test;

† -Bilaterally operated cases first eye surgery date was taken for calculation of duration

Based on presenting [Table 3] and the best-corrected visual acuity [Table 4], there was a significant difference between aphakic and pseudophakic eyes. Pseudophakic eyes had better visual acuity outcomes than aphakic eyes. On presentation, a smaller proportion of aphakic eyes had visual acuity of 20/20 (Odds ratio 0.1, 95% CI 0.03 to 0.24%) when compared to pseudophakics. Aphakics had 2.95 times higher odds of having visual acuity less than 20/60 when compared to pseudophakics [Table 3]. Even with best refractive correction only a small proportion of aphakic eyes (6.9%) had a visual acuity of 20/20. When compared to pseudophakics, aphakics had 3.67 times higher odds of having visual acuity less than 20/60, even with best refractive correction [Table 4]. This suggests that pseudophakics had better visual outcome after cataract surgery. Table 5 provides data on presenting visual acuity for the subjects in the better eye.

**Table 3:** Presenting visual acuity status in aphakic and pseudophakic eyes†

Visual acuity	Aphakia			Pseudophakia			*P* value (OR, 95% CI)
			
	Rural*	Urban	Total	Rural*	Urban	Total	
20/20	0	5 (3.0)	5 (1.0)	20 (4.7)	61(13.9)	81 (9.4)	<0.001 (0.10, 0.03 to 0.24)
20/25 to 20/60	105 (30.7)	80 (48.8)	185 (36.6)	205 (48.1)	268 (60.9)	473 (54.6)	<0.001 (0.47, 0.38 to 0.60)
Less than 20/60	237 (69.3)	79 (48.2)	316 (62.5)	201(47.18)	111 (25.2)	312 (36.0)	<0.001 (2.95, 2.35 to 3.70)
Less than 20/200	141 (41.2)	50 (30.5)	191 (37.7)	65 (15.3)	36 (8.2)	101 (11.7)	<0.001 (4.59, 3.49 to 6.04)
Less than 20/400	112 (32.7)	47 (28.7)	159 (31.4)	46 (10.8)	27 (6.1)	73 (8.4)	<0.001 (4.98, 3.67 to 6.75)
Total number of eyes in the study	342	164	506	426	440	866	

* -In 13 eyes the anterior segment details could not be visualized due to the gross distortion of the eyeball anatomy following cataract surgery;

† -Chi square test

**Table 4:** Best-corrected visual acuity status in aphakic and pseudophakic eyes†

Visual acuity	Aphakia			pseudophakia			*P* value (OR, 95% CI)
			
	Rural*	Urban	Total	Rural*	Urban	Total	
20/20	9 (2.6)	26 (15.9)	35 (6.9)	60 (14.1)	178 (40.5)	238 (27.5)	<0.001 (0.20, 0.13 to 0.29)
20/25 to 20/60	200 (58.5)	89 (54.3)	289 (57.1)	296 (69.5)	217 (49.3)	513 (59.2)	0.47 (0.91, 0.73 to 1.14)
Less than 20/60	133 (38.9)	49 (29.9)	182 (36.0)	70 (16.4)	45 (10.2)	115 (13.3)	<0.001 (3.67, 2.80 to 4.79)
Less than 20/200	76 (22.2)	32 (19.5)	108 (21.3)	37 (8.7)	27 (6.1)	64 (7.4)	<0.001 (3.40, 2.44 to 4.74)
Less than 20/400	54 (15.8)	29 (17.7)	83 (16.4)	29 (6.8)	22 (5.0)	51 (5.9)	<0.001 (3.13, 2.17 to 4.53)
Total number of eyes	342	164	506	426	440	866	

* -In 13 eyes the anterior segment details could not be visualized due to the gross distortion of the eyeball anatomy following cataract surgery;

† -Chi square test

**Table 5:** Visual acuity in better eye of cataract-operated subjects

Visual acuity in better eye	Presenting visual acuity (%)		Corrected visual acuity (%)	
		
	Rural	Urban	Rural	Urban
20/20	16 (3.0)	65 (16.0)	63 (11.9)	181 (44.6)
20/25 to 20/60	260 (49.2)	265 (65.3)	379 (71.8)	203 (50.0)
Less than 20/60	252 (47.7)	76 (18.7)	86 (16.3)	22 (5.4)
Less than 20/200	29 (5.7)	6 (1.5)	11 (2.1)	6 (1.5)
Less than 20/400	57 (11.1)	17 (4.2)	25 (4.7)	5 (1.2)
Total	528	406	528	406

Table 6 lists the causes for visual impairment. Uncorrected refraction was the major cause of visual impairment in both the pseudophakic and aphakic groups. Following refractive correction, cystoid macular edema (CME) was the major cause of visual impairment in the aphakic group. In the pseudophakic group the causes of visual impairment were posterior capsule opacification (PCO), retinal pathology and corneal pathology. Those with aphakia were more likely (OR 1.8; 95% CI 1.3 to 2.6%) to have visual acuity of <20/60 to ≤20/400 or visual acuity of <20/400 (OR 6.2; 95% 4.0 to 9.8%). The rural cataract-operated population was also more likely (OR 3.2; 95% CI 2.2 to 4.5%) to have visual acuity of <20/60 to ≤20/400 and visual acuity of <20/400 (OR 3.5; 95% CI 2.3 to 5.5%) [Table 7].

**Table 6:** Causes for visual impairment following cataract surgery in rural and urban population

Causes for visual impairment	Visal acuity (<20/60 to ≤20/400)Number of eyes (%)(Rural*, Urban)		Visual acuity (<20/400)Number of eyes (%)(Rural*, Urban)	
		
	Pseudophakia	Aphakia	Pseudophakia	Aphakia
Uncorrected refractive error	175 (73.2) (114,61)	58 (36.9) (46,12)	22 (30.1) (17,5)	76 (47.2) (58,18)
Retinal pathology	6 (2.5) (3,3)	15 (9.6) (12,3)	14 (19.2) (5,9)	14 (8.7) (9,5)
Corneal pathology	3 (1.3) (2,1)	3 (1.9) (2,1)	9 (12.3) (7,2)	19 (11.8) (14,5)
Posterior capsule opacification	28 (11.7) (19,9)	3 (1.9) (2,1)	11 (15.1) (9,2)	8 (5.0) (4,4)
Glaucoma	5 (2.1) (1,4)	6 (3.8) (4,2)	6 (8.2) (3,3)	11 (6.8) (9,2)
Cystoid macular edema	12 (5.0) (7,5)	37 (23.6) (31,6)	Nil	7 (4.3) (5,2)
Optic atrophy	3 (1.3) (3,0)	5 (3.2) (5,0)	3 (4.1) (1,2)	12 (7.5) (8,4)
Unexplained	1 (0.4) (1,0)	4 (2.5) (4,0)	Nil	6 (3.7) (4,2)
Others	Nil	6 (3.8) (2,4)	4 (5.5) (2,2)	2 (1.2) (0,2)
Dull foveal reflex	2 (0.8) (1,1)	14 (8.9) (12,2)	2 (2.7) (1,1)	3 (1.9) (3,0)
Poor cataract surgery	4 (1.7) 4,0)	6 (3.8) (5,1)	2 (2.7) (1,1)	3 (1.9) (0,3)
Total	239	157	73	161

* -In 13 eyes of rural population the anterior segment details could not be visualized due to the gross distortion of the eyeball anatomy following cataract surgery

**Table 7:** Multiple logistic regression for risk factors for visual impairment in cataract-operated eyes

	Visual acuity (<20/60 to ≤20/400) odds ratio (95% CI)	Visual acuity (<20/400) odds ratio (95% CI)
Age group		
40-49	1	1
50-59	2.5 (1.0 to 6.5)	1.87 (0.6 to 5.6)
60-69	1.5 (0.6 to 3.7)	0.94 (0.3 to 2.7)
70-79	2.0 (0.8 to 4.8)	2.10 (0.8 to 5.8)
80 and above	2.2 (0.7 to 6.6)	2.5 (0.7 to 8.7)
Male	1	1
Female	1.4 (1.0 to 2.0)	1.0 (0.7 to 1.5)
Urban population	1	1
Rural population	3.2 (2.2 to 4.5)	3.5 (2.3 to 5.5)
Duration from surgery		
<3 years	1	1
≥3 years	1.0 (0.7 to 1.4)	1.2 (0.8 to 1.8)
Pseudophakia	1	1
Aphakia	1.8 (1.3 to 2.6)	6.2 (4.0 to 9.8)
Literate	1	1
Illiterate	1.8 (1.3 to 2.5)	1.4 (0.9 to 2.1)

CI - Confidence interval

## Discussion

The rural study population was derived from two districts in the northern part of Tamil Nadu. The rural study area is located about 65 kilometers from Chennai city; in addition they had access to vision care in the nearby district headquarter hospitals. The urban study population was from a city with an estimated 400 ophthalmologists. According to the National survey on blindness and visual outcomes after cataract surgery in individuals aged 50 years and older, the overall surgical coverage in Tamil Nadu was 82.8% and the cataract surgical rate was 14.7%.[15] In terms of numerical surgical performance the state was amongst the top five states in the 15 states studied.

The prevalence of cataract surgery in subjects aged 40 years and above was 13.5% (95% CI 12.4-14.6%) in our rural study population and 10.5% (95% CI 9.6 -11.5%) in the urban population. Estimates were done for 50 years and above in other population-based studies and the national survey.[8–10,15] When we analyzed the subset of 50 years and above our cataract surgery rates were 21.9% for the rural study group and 15.7% for the urban group. There seem to be differences in the cataract surgery prevalence rates and outcomes reported by the various studies. These differences are partially due to the varying definitions of blindness used in the studies, the period of study and varying intervals between measurement of visual acuity and the cataract surgery.[16]

In the present study, good outcomes at presentation based upon World Health Organization (WHO) definitions[17] (visual acuity of ≥20/60) were seen in 42.3% (330 eyes) of the rural and 68.5% (414 eyes) of the urban cataract-operated. This improved to 72.3% (565 eyes) and 84.4% (510 eyes), respectively, with appropriate refraction, suggesting that among 30% of rural subjects and 16% of urban subjects who had undergone cataract surgery, the cause for poor or very poor visual outcome was non-use of spectacles or improper refraction following cataract surgery. A similar trend was seen when we analyzed pseudophakes and aphakes separately [Tables 3 and 4]. Our findings reemphasize those reported by the other studies, reiterating the need for appropriate refraction and spectacle prescription following cataract surgery.[8–10] Poor outcomes due to uncorrected refraction will remain a major cause for poor visual outcome after cataract surgery, unless this is addressed.

Small-incision cataract surgical techniques have lower induced astigmatism than conventional extra-capsular cataract extraction, and are becoming increasingly popular.[18] With increased penetration of these surgical techniques, it is possible, that the number of those with visual impairment due to uncorrected refractive error will decline. The surgical technique and choice of IOL inserted should be tailored to individual patient parameters and refractive needs (as determined from a good history, a comprehensive eye examination and appropriate IOL power measurements) and should aim for near emmetropia postoperatively in the majority of cases.

In our rural subjects 55.4% cataract-operated eyes had an IOL implanted. After excluding the 40-49 years age group this was 57.7%. This is less than the 63.0% pseudophakia among individuals aged 50 years or more reported by Nirmalan *et al*. from the same state.[10] The study was conducted during the same time period in a rural population residing in a southern district of Tamil Nadu. One possible reason for their higher pseudophakia rate in their study could be due to the fact that their study area was a reputed non-governmental organization hospital with dedicated trained ophthalmologists. In contrast to this, our pseudophakia rate was much higher than the reported 5.8% pseudophakia in a rural population aged 50 years and more in Rajasthan for a study conducted in the late 1990s.[9] Their low rate was related to the technique followed; in their population 94.2% were aphakics and 92% had planned intracapsular cataract extraction. The authors highlighted that the causes were mainly inadequate facilities, lack of skilled surgeons and non-availability of IOLs. In our urban population, 72.8% were pseudophakics and this proportion is significantly higher than that for the rural population. This difference could be related to better facilities for IOL implantation in our urban area. Our reported urban pseudophakia rate is higher than the 42% of pseudophakia reported from an urban population in the Andhra Pradesh Eye Diseases Study.[8] One reason for this could be the six-year time interval between the studies, the other reason could be probably due to the differences in the availability of eye care services in the study areas.

When we compared outcomes of pseudophakia and aphakia, pseudophakes had significantly better outcomes than aphakes. With refractive error correction, 83.6% rural pseudophakes and 89.8% of urban pseudophakes had visual acuity of ≥20/60, in contrast to only 61.1% and 70.1% of aphakes respectively (*P* < 0.001). Aphakes and those residing in the rural study area were significantly more likely to have visual impairment. The major lacuna in our study was lack of availability of surgical data. It was difficult to predict whether the aphakia was due to planned intracapsular surgery or due to a complication of extracapsular cataract surgery. If aphakia was due to complicated extracapsular surgery, the complication would have contributed to the visual acuity of <20/60. In spite of this drawback in the study we can infer from our data that extracapsular cataract surgery with IOL insertion, performed well and followed by providing appropriate refractive correction can yield visual acuity of ≥20/60 in more than 80% of both rural and urban residents.

Surgery-related problems were responsible for visual impairment in both the pseudophakic and aphakic groups. In the pseudophakic group, the main cause of visual impairment was PCO. The causes of PCO are multifactorial.[19] Technique of the surgery, type of IOL used, and postoperative inflammation are some of the factors. From our data we could not identify the causes of PCO in our population. Improvement in surgical technique and appropriate postoperative follow-up could reduce the chances of PCO. In the aphakia group the main cause was CME, since CME is related to surgery and can be prevented/reduced with improved surgical technique.

In our study, the visual outcomes after cataract surgery were better in eyes with pseudophakia. Uncorrected refraction, surgery-related complications such as PCO and CME were the major causes for poor outcomes. The goal of the national program is to perform 80% of the cataract surgeries with IOLs. It is encouraging to know that this target can be achieved with improved training programs and strengthening of the eye care infrastructure.[7] WHO has provided guidelines for cataract surgery outcomes- 85% should have visual acuity of 20/60 or better, 10% have less than 20/60-20/200 and less than 5% should have a visual acuity of less than 20/200.[20] Our rural study population falls short of these guidelines, but our urban outcomes are very close to WHO recommendations. Cataract outcomes can be definitely improved with a good follow-up component in the cataract blindness program that results in elimination of the treatable causes for poor outcomes. Though the proportion of IOL implant surgery has increased, support services such as the availability of YAG lasers and infrastructure for follow-up have not kept pace. There is a need to enhance the cataract surgery program to include adequate infrastructure for postoperative monitoring and appropriate management. By improving this facility, the prevalence of visual impairment in pseudophakics can be minimized.
